# Detecting the differential genomic variants using cross-population phenotype-associated variant (XP-PAV) of the Landrace and Yorkshire pigs in Korea

**DOI:** 10.1080/19768354.2021.2006310

**Published:** 2021-11-29

**Authors:** Young-Sup Lee, Seungwoo Son, Jaeyoung Heo, Donghyun Shin

**Affiliations:** a Department of Animal Biotechnology, Jeonbuk National University, Jeonju, Republic of Korea; b Department of Agricultural Convergence Technology, Jeonbuk National University, Jeonju, Republic of Korea

**Keywords:** Cross-population phenotype associated variant (XP-PAV), differential genomic variant, t-test, back fat thickness, daily weight gain

## Abstract

Although there have been many genome-wide association studies (GWAS) and selective sweep analyses to understand pig genomic regions related to growth performance, these methods considered only the gene effect and selection signal, respectively. In this study, we suggest the cross-population phenotype associated variant (XP-PAV) analysis as a novel method to determine the genomic variants with different effects between the two populations. XP-PAV analysis could reveal the differential genetic variants between the two populations by considering the gene effect and selection signal simultaneously. In this study, we used daily weight gain (DWG) and back fat thickness (BF) as phenotypes and the Landrace and Yorkshire populations were used for XP-PAV analysis. The main aim was to reveal the differential selection by considering the gene effect between Landrace and Yorkshire pigs. In the gene ontology analysis of XP-PAV results, differential selective genes in DWG analysis were involved in the regulation of interleukin-2 production and cell cycle G2/M transition. The protein modification and glycerophospholipid biosynthetic processes were the most enriched terms in the BF analysis. Therefore, we could identify genetic differences for immune and several metabolic pathways between Landrace and Yorkshire breeds using the XP-PAV analysis. In this study, we expect that XP-PAV analysis will play a role in determining useful selective variants with gene effects and provide a new interpretation of the genetic differences between the two populations.

## Introduction

Swine is one of the most important livestock and a major protein source for humans (Amaral et al. 2011; Wilkinson et al. 2013). The domesticated pig originated from the wild boar (*Sus scrofa*). Many pig breeds have been selected for desirable traits such as rapid growth, increased lean meat, and enhanced prolificacy, and multiple studies have investigated the traits relevant to the growth and developmental processes. Daily weight gain (DWG) and back fat thickness (BF) are important economic traits with regards to growth performance in pig breeding (Malek et al. 2001). Pigs have also been bred according to the species characteristics. One among the preferred breeds, the Landrace (LL) pigs originate from Denmark; the word Landrace means ‘native species’ The pigs of this breed are large with good growth, have a large body weight, and body length and depth. Pigs of the Yorkshire (YY) breed have a large and well-developed body, good growth rate, and their overall shape is rectangular.

From a genomic perspective, identification and characterization of selection signatures were used to examine the genetic basis for phenotypic variations in swine (Andersson and Georges 2004). Previous genome-wide association studies (GWAS) focused on the examination of significant variants and evolutionary genomic analysis to determine the selective genomic regions by comparing the two breeds. Cross-population extended haplotype homozygosity (XP-EHH), cross-population composite likelihood ratio (XP-CLR), and hapFLK were used as selective sweep study methods in these studies (Manunza et al. 2016; Sun et al. 2018; Wang et al. 2020). When a beneficial mutation arises and subsequently spreads in the genome, a selective sweep occurs which generates higher population differentiation, higher frequencies of segregating sites, and linkage disequilibrium (LD) (Grossman et al. 2010). The XP-EHH test detects the occurrence of selection based on LD whereas the XP-CLR test considers the spatial patterns of allele frequencies of single nucleotide polymorphisms (SNPs) (Chen et al. 2010). Both XP-CLR and XP-EHH are frequently used to compare the genomic differences and signals of selection between two populations. However, the selective sweep analysis only considers the allele configuration and homozygous genomic regions, excluding the traits, and GWAS only considers the significant variants associated with the traits. Similarly, previous selective sweep and genome wide association (GWA) studies focused on the change of allele constitutions around the beneficial alleles and the association with the phenotypic values, respectively. Therefore, we suggest cross-population phenotype-associated variants (XP-PAV) as a novel method to identify selective genetic variants with meaningful gene effects between the two populations. This method could consider both the variant effect and allele configuration (VanRaden et al. 2017). It could also determine the significant variants containing selective signals and gene effects related to each phenotype.

In the previous study, selective sweeps between pig breeds were explored using XP-CLR and XP-EHH (Jeong et al. 2015; Arora et al. 2021). They found the variants and the genes showing the selective sweeps. The results in XP-CLR and XP-EHH, in which the variants and the genes were involved can be putative differential genomic regions between compared breeds. Meanwhile, RNA-seq usually determines differential expressed genes (DEGs) between two groups having a difference for an interesting phenotype (Jung et al. 2020). In this study, we tried to determine the differential genomic variants between two breeds. We used the LL and YY populations for XP-PAV analysis and performed genome analysis using DWG and BF as the targeted phenotypes in each population. The effect difference between LL and YY breeds was tested. Gene ontology (GO) analysis of XP-PAV results revealed that the differential selective genes related to DWG were involved in the regulation of interleukin-2 production and cell cycle G2/M transition, whereas the genes related to BF were associated with the protein modification and glycerophospholipid biosynthetic processes. Based on the results of the XP-PAV analysis between LL and YY breeds, we identified genetic differences in immune response, lipid metabolism, and protein modification processes. Our study attempted to examine the role that XP-PAV analysis could play in determining the useful selective variants with gene effects in the future and provide a new interpretation of the genetic differences between the two populations.

## Materials and methods

### Data preparation and examination

The study protocols and standard operating procedures of the pig samples were reviewed and approved by the Institutional Animal Care and Use Committee of the National Institute of Animal Science. The 3,356 LL pigs and 6,965 YY pigs were sampled from the Great Grand Parent (GGP) farms. The DWG was measured from birth to the end of the test. The average value of BF was calculated by taking a length of 5 cm from the midline to the left or right of the three parts–the shoulder (the fourth rib), back (the last rib), and waist (the last lumbar vertebra). The genomic DNA of the pigs was genotyped using the Illumina Porcine 60 K SNP BeadChip (Illumina, San Diego, CA, USA). A total of 61,565 genotyped SNPs were filtered using quality control with minor allele frequency (MAF, <0.05), and missing data (>0.05), which retained 40,078 and 41,608 SNPs in LL and YY pigs, respectively. The genotypes were imputed using Beagle 5.1 (Browning and Browning 2007). The number of SNPs used after QC in the XP-PAV analysis was 44,273.

To determine the differentially significant variants between the two populations, population structure analysis should be performed. The utility of the XP-PAV analysis between the LL and YY pigs’ data had to be assessed. A PCA was performed to check the genetic dissimilarity between the two breeds. The principal components, PC1 and PC2, demonstrated differences between the two populations. For PCA, we used the GCTA program (Yang et al. 2011).

### XP-PAV analysis

To obtain the marker’s beta effect, we performed a GWA test for the LL and YY populations. In this analysis, the beta effect is the slope coefficient of the linear model. The phenotypes used were DWG and BF, and the covariates were sex and parity in the GWA test. The results of the GWA test were utilised for further analysis. In XP-PAV analysis, we aimed to determine the differential selective genetic variant, considering the marker effects between the two breeds. GWA tests showed differences in marker effects between the two breeds. We considered the marker effects and allele configuration at the given marker using the XP-PAV method model.

(1) $$the\;components\;of\;LL_{j}\in set(\left\{0,\;1,\;2\right\})$$

(2) $$the\;components\;of\;YY_{j}\in set(\left\{0,\;1,\;2\right\})$$

(3) $$LL_{j}=\;\left[\begin{array}{c} \begin{array}{c} Indiv1\;allele\;coding\;:0\\ \begin{array}{c} Indiv2\;allele\;coding\;:1\\ \begin{array}{c} Indiv3\;allele\;coding\;:2\\ \begin{array}{c} Indiv4\;allele\;coding\;:0\\ \ldots \end{array} \end{array} \end{array} \end{array}\\ Indiv3356\;allele\;coding\;:0 \end{array}\right],\;\;YY_{j}=\;\left[\begin{array}{c} \begin{array}{c} Indiv1\;allele\;coding\;:0\\ \begin{array}{c} Indiv2\;allele\;coding\;:1\\ \begin{array}{c} Indiv3\;allele\;coding\;:2\\ \begin{array}{c} Indiv4\;allele\;coding\;:0\\ \ldots \end{array} \end{array} \end{array} \end{array}\\ Indiv6965\;allele\;coding\;:0 \end{array}\right]$$

(4) $$t.test(LL_{j}u,YY_{j}v)$$

where u and v are j SNP effects in the LL and YY pigs, respectively. In the Equations (1) and (2), each vector component of $LL_{j}$ and $YY_{j}$ belongs to the set of the additive allele coding. In the Equation (3) demonstrates the allele coding in j SNP. In the t-test, $LL_{j}*$u represents the vector that constitutes the LL individuals allele coding ([indiv1 allele coding, indiv2 allele coding, … .,indiv 3356 allele coding] = [0,1,2,0, … .,0]) multiplied by the GWAS beta effect (in this formula, u) in SNP j. $YY_{j}*v$ is represented similarly (Equation 4). By considering the GWA test results and allele configuration at the same time, the significant variants in XP-PAV denoted the differential variants between the two breeds with respect to the associated traits. As seen in Equation (4), the t-test was performed for each marker for XP-PAV analysis. The sample size was large enough to test the mean difference in the effect. However, we observed a very low *p*-value due to the repetition of three canonical points (0, 1, 2 as seen in (1) and (2)) and large sample sizes. Hence, we standardized the t-values for obtaining reasonable *p*-values. Statistical significance was set at *p* < 0.05, and putative candidate genes in each DWG and BF were considered significant. Low *p*-values of genes indicate that they differ between LL and YY pigs at the variant level.

### GO analysis

To identify the biological significance of the genetic variants in the XP-PAV test, we performed GO analysis for genes related to significant SNPs using the Database for Annotation, Visualization, and Integrated Discovery (DAVID) v6.8 program (https://david.ncifcrf.gov). The DAVID tool provides a comprehensive set of functional annotations to understand the biological significance of a large list of genes. The significant markers obtained from the XP-PAV analysis were matched to the gene information using the Ensemble website (www.ensembl.org) (reference genome version: Sus scrofa 11.1).

## Results

### Data description

**Figure 1.**
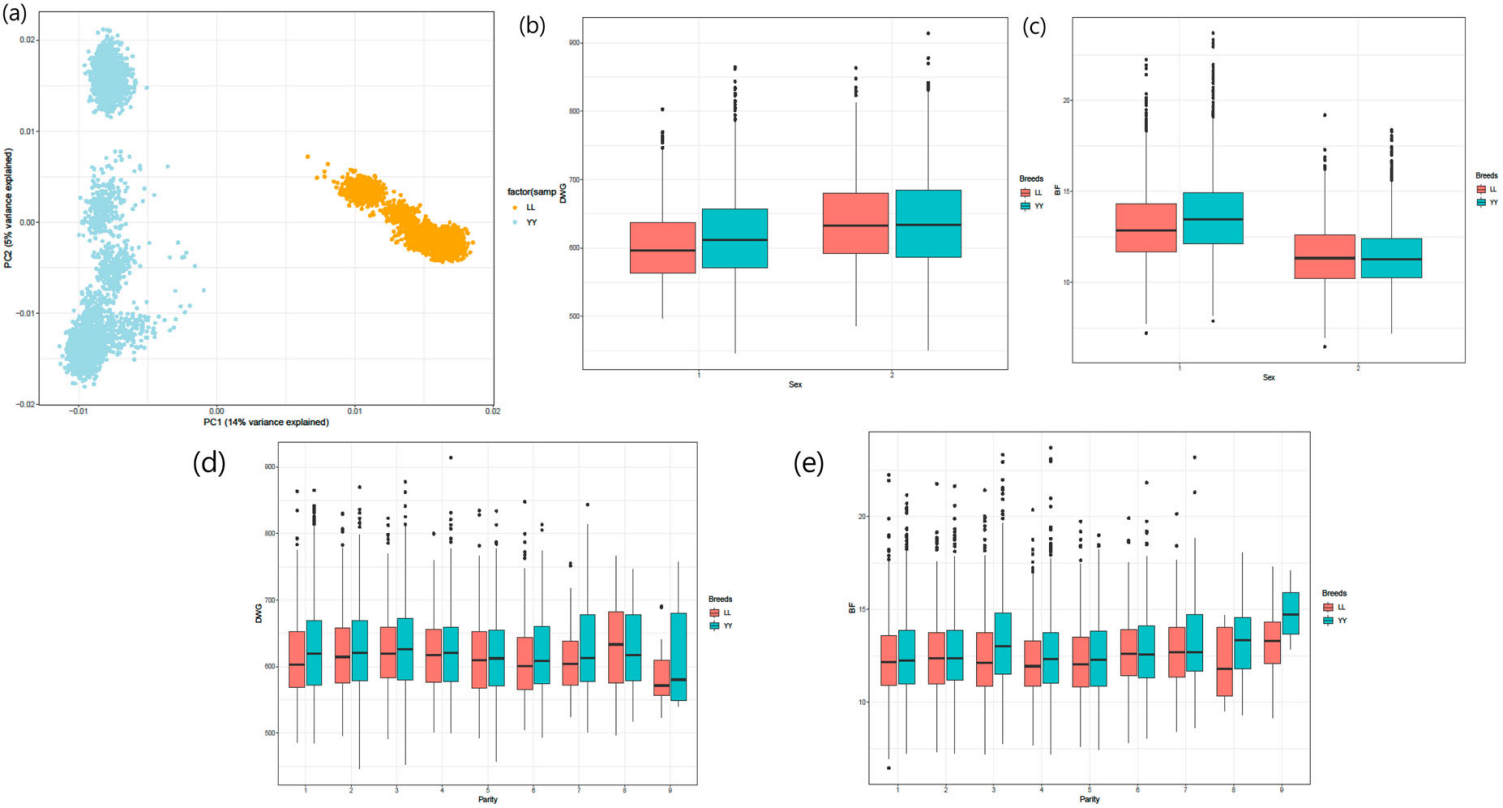
(a) Principal Component Analysis (PCA) plot of Landrace (LL) and Yorkshire (YY) pigs. By PC1, two breeds were distinct. (b, c, d, e) The boxplot according to sex (b, c) and parity (d, e) in LL and YY, respectively. The boxplot of sex and parity did not show a significant difference between LL and YY pigs, but difference in marker effects in genome-wide association (GWA) test was observed.

The average (± standard deviation) of SNP distances ranged from chromosome 14: 39,318 bp (± 639) to chromosome 13: 53,343 bp (± 998) in both LL and YY datasets. The PCA demonstrated the genetic distinction between LL and YY, especially in PC1 (total variance 14% explained) and PC2 (total variance 5% explained) (Figure. 1a). The boxplots in LL and YY showed global trends in sex and parity, which were used as covariates in the GWA study (Figure 1b and c).

### XP-PAV analysis result

GWA tests have been used to identify putative variants and corresponding candidate genes associated with these phenotypes. In previous studies, DWG and BF have been the phenotypes of interest to evaluate several growth traits for GWA tests in pig breeding (Do et al. 2014; Guo et al. 2017). In the two pig populations, the calculated effects from the GWA test for each SNP were not the same, and the allele configurations of each population were different. Therefore, after the GWA test was conducted for each population, the different marker effects as well as allele configurations were taken into account in comparative genomics analysis. In the XP-PAV results, we found that the significant SNPs (XP-PAV test *p*-value <0.05) were 1,259 and 1,065 in DWG and BF, respectively (Figure. 3).

**Figure 2.**
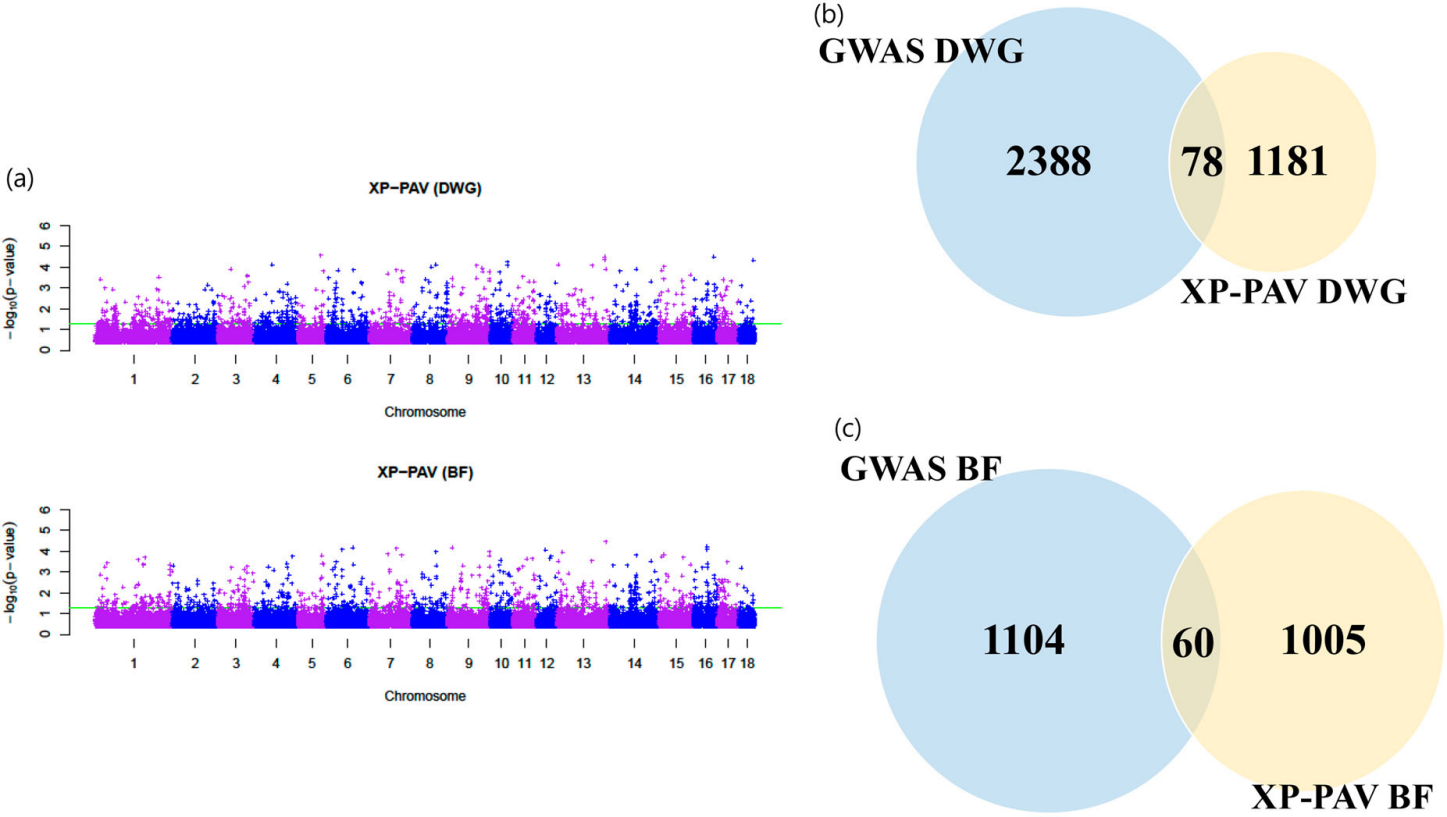
(a) Manhattan plot of XP-PAV results of –log10(*p*-value). (b, c) The Venn diagram of results of the number of significant variants in genome-wide association studies (GWAS) and cross-population phenotype associated variant (XP-PAV). The significant variants’ *p*-values were Bonferroni *p*-value <0.01 in GWAS daily weight gain (DWG), *p*-value <0.001 in GWAS back fat thickness (BF), and *p*-value <0.05 in XP-PVA DWG and BF, respectively.

**Figure 3.**
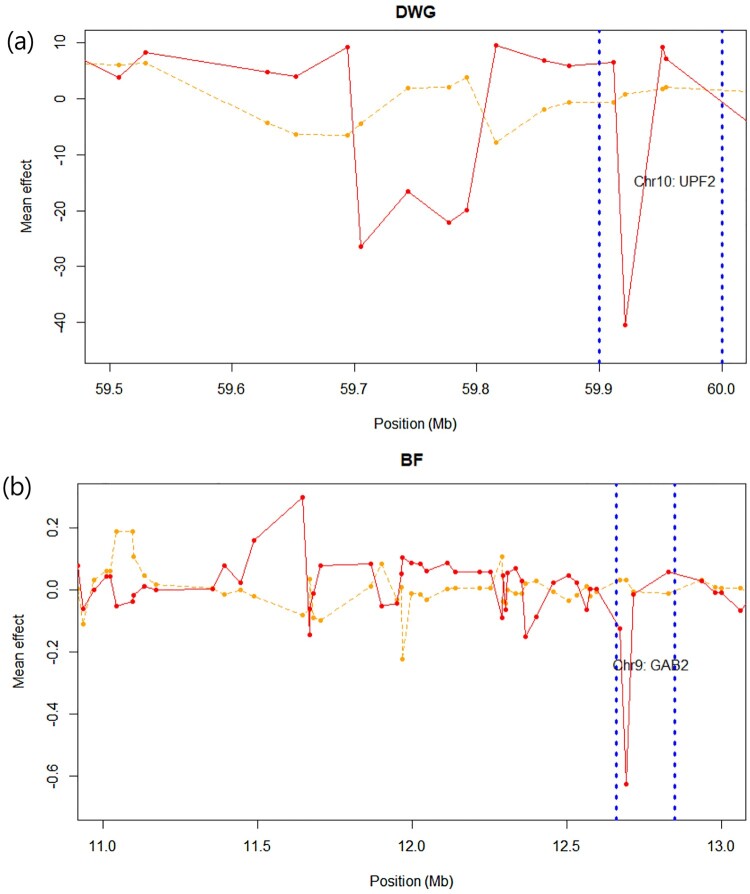
The mean effect plot of Landrace (LL; orange) and YY (Yorkshire; red). The gene regions with lowest *p*-value were presented in blue lines. (a) In daily weight gain (DWG), the regulator of nonsense mediated mRNA decay (UPF2) was highly significant. (b) In back fat (BF), GRB2 associated binding protein 2 (GAB2) was the most significant.

Figure 2a demonstrates (–1)*log_10_(*p*-value) of the XP-PAV results in DWG and BF, respectively. Figure 2b and c show the Venn diagram of the number of significant variants in the GWA study and XP-PAV (GWAS DWG: Bonferroni *p*-value <0.01, GWAS BF: *p*-value <0.001, and XP-PAV in both phenotypes *p*-value <0.05). Figure 3a and b demonstrate the difference in the mean effect of LL and YY (orange: LL, red: YY) pigs in the genomic regions and the most significant genes (UPF2: *p*-value 5.48E-05 and GAB2: *p*-value 6.96E-05) (Table 1). The mean effect of the variant was from the mean value of $LL_{j}$ and $YY_{j}$ as shown in Equations (1) and (2), respectively. In the diagram, the significant genes were highly differentially affected genes. Regulators of nonsense-mediated mRNA decay (UPF2) and GRB2-associated binding protein (GAB2) are shown in Figure 3. UPF2 encodes a protein that is part of a post-splicing multiprotein complex and is involved in both mRNA nuclear export and mRNA surveillance. mRNA surveillance plays a role in detecting exported mRNAs with truncated open reading frames and initiates nonsense-mediated mRNA decay (NMD). GAB2 protein acts as an adapter for transmitting various signals in response to stimuli through growth factor receptors, cytokines, and T- and B-cell antigen receptors (www.genecards.org). Table 1 shows the highly significant SNPs and those encompassing genes in the XP-PAV test. Genes related to SNPs (*p* < 0.05) in XP-PAV were selected and used for the GO analysis. In DWG, the regulation of interleukin-2 production and cell cycle G2/M transition were the most enriched terms (Table 2). The glycerophospholipid biosynthetic and protein modification processes were the most enriched terms in BF (Table 3) (Rule et al. 1989).

**Table 1.** The significant detected single nucleotide polymorphisms (SNPs) (top 5 in each phenotype) and encompassing genes in cross-population phenotype-associated variant (XP-PAV)

CHR	SNP	Position	*p*-value	Genes	Phenotypes
13	ASGA0059801	203,697,839	3.46E-05	*DSCAM*	BF
9	ALGA0051430	12,691,365	6.96E-05	*GAB2*	BF
16	ALGA0090605	48,859,252	7.99E-05	*ZNF366*	BF
6	MARC0018856	65,196,951	8.17E-05	*TP73*	BF
16	ASGA0073290	48,791,895	8.17E-05	*ZNF366*	BF
13	ALGA0073375	198,378,920	3.12E-05	*RUNX1*	DWG
10	ASGA0102557	59,920,758	5.48E-05	*UPF2*	DWG
4	ALGA0025364	67,304,102	7.90E-05	*CPA6*	DWG
10	ALGA0059370	60,133,955	8.02E-05	*ECHDC3*	DWG
13	ALGA0071935	137,841,013	8.25E-05	*PARP14*	DWG

**Table 2.** Gene Ontology (GO) of differential genes in daily weight gain (DWG) (cross-population phenotype-associated variant; XP-PAV *p*-value <0.05). The most enriched terms were regulation of interleukin-2 production and G2/M cell cycle.

Term	Count	*p*-value	Genes	Fold enrichment
GO:0032743∼positive regulation of interleukin-2 production	5	0.000	*GLMN, PDE4B, VTCN1, MAP3K7, RUNX1*	14.2
GO:1902882∼regulation of response to oxidative stress	6	0.000	*FUT8, VNN1, FBXW7, PSAP, UBQLN1, STOX1*	9.0
GO:0044839∼cell cycle G2/M phase transition	7	0.001	*CENPF, ENSA, CDK1, PLCB1, KAT14, FBXL7, STOX1*	6.0
GO:0032663∼regulation of interleukin-2 production	5	0.002	*GLMN, PDE4B, VTCN1, MAP3K7, RUNX1*	9.0
GO:0050801∼ion homeostasis	17	0.002	*PTGFR, GCM1, CAMK2D, CCL21, NEDD4L, PSEN1, CP, ESR1, DIAPH1, STIM1, SLC9A9, TAC4, ANXA7, RHCG, CCL28, CORIN, MCU*	2.4
GO:0000086∼G2/M transition of mitotic cell cycle	6	0.003	*ENSA, CDK1, PLCB1, KAT14, FBXL7, STOX1*	6.2
GO:1900407∼regulation of cellular response to oxidative stress	5	0.003	*FUT8, VNN1, FBXW7, PSAP, UBQLN1*	8.4
GO:0098655∼cation transmembrane transport	14	0.003	*CAMK2D, CCL21, KCNIP4, SLC39A11, NEDD4L, DIAPH1, STIM1, KCNMA1, UBQLN1, RHCG, CACNG2, ATP6V1C1, MCU, ATP6V1C2*	2.6
GO:0044770∼cell cycle phase transition	11	0.003	*CENPF, CAMK2D, ENSA, CDK1, NEK10, ATM, PLCB1, KAT14, FBXL7, CDC14A, STOX1*	3.1
GO:0032623∼interleukin-2 production	5	0.003	*GLMN, PDE4B, VTCN1, MAP3K7, RUNX1*	7.9
GO:0045785∼positive regulation of cell adhesion	11	0.004	*VNN1, MYO10, CCL21, KIF26B, ALOX15, VWC2, VTCN1, CD47, CCL28, VAV1, SKAP1*	3.0

**Table 3.** Gene Ontology (GO) of differential genes in back fat thickness (BF) (cross-population phenotype-associated variant; XP-PAV *p*-value <0.05). The most enriched terms were the protein modification and glycerolipid biosynthetic processes.

Term	Count	*p*-value	Genes	Fold Enrichment
GO:0046474∼glycerophospholipid biosynthetic process	6	0.001	ALOX15, PIGU, PIGK, ATM, SLC27A1, CRLS1	7.6
GO:0036211∼protein modification crprocess	47	0.001	TSSK1B, ALOX15, ARFGEF1, UBE2 K, NEK10, SATB1, NAA16, FBXW7, MEF2C, ITGA5, STT3B, PBLD, DAB2, MAST1, PRKAR2A, KAT7, SLC27A1, ASXL1, RNF13, P4HA1, PIGU, FBXO21, CAMK2D, MEF2A, PLCB1, WWTR1, TBC1D7, JMJD1C, PSAP, ZDHHC1, STOX1, RNF144B, ATM, CCL21, ZER1, KAT14, UBQLN1, ATG7, TNFRSF19, PIGK, KLHL29, GCLC, NEDD4L, KLHL1, USP54, ENSA, HLTF	1.6
GO:0006464∼cellular protein modification process	47	0.001	TSSK1B, ALOX15, ARFGEF1, UBE2 K, NEK10, SATB1, NAA16, FBXW7, MEF2C, ITGA5, STT3B, PBLD, DAB2, MAST1, PRKAR2A, KAT7, SLC27A1, ASXL1, RNF13, P4HA1, PIGU, FBXO21, CAMK2D, MEF2A, PLCB1, WWTR1, TBC1D7, JMJD1C, PSAP, ZDHHC1, STOX1, RNF144B, ATM, CCL21, ZER1, KAT14, UBQLN1, ATG7, TNFRSF19, PIGK, KLHL29, GCLC, NEDD4L, KLHL1, USP54, ENSA, HLTF	1.6
GO:0007010∼cytoskeleton organization	22	0.002	ALOX15, PHACTR1, TACC2, MAP2, MEF2A, DST, MYPN, ARHGAP10, ARFGEF1, DOCK1, CCL21, AP1AR, EPB41L2, CDC42EP4, MEF2C, CDC14A, VPS54, KIF23, TPPP3, MAST1, ARHGAP26, SVIL	2.1
GO:0032446∼protein modification by small protein conjugation	15	0.002	RNF13, FBXO21, WWTR1, TBC1D7, RNF144B, UBE2 K, FBXW7, ZER1, UBQLN1, ATG7, NEDD4L, KLHL29, GCLC, KLHL1, HLTF	2.5
GO:0070647∼protein modification by small protein conjugation or removal	17	0.002	ASXL1, RNF13, FBXO21, WWTR1, TBC1D7, RNF144B, UBE2 K, FBXW7, ZER1, UBQLN1, ATG7, NEDD4L, KLHL29, GCLC, KLHL1, USP54, HLTF	2.3
GO:0071277∼cellular response to calcium ion	4	0.003	CARF, ALOX15, MEF2C, MEF2A	13.5
GO:0016567∼protein ubiquitination	14	0.003	RNF13, FBXO21, WWTR1, TBC1D7, RNF144B, UBE2 K, FBXW7, ZER1, UBQLN1, NEDD4L, KLHL29, GCLC, KLHL1, HLTF	2.6
GO:0045017∼glycerolipid biosynthetic process	6	0.004	ALOX15, PIGU, PIGK, ATM, SLC27A1, CRLS1	5.8
GO:0008654∼phospholipid biosynthetic process	6	0.004	ALOX15, PIGU, PIGK, ATM, SLC27A1, CRLS1	5.7
GO:0006650∼glycerophospholipid metabolic process	7	0.005	ALOX15, PIGU, PIGK, ATM, PIK3CD, SLC27A1, CRLS1	4.5

## Discussion

The extant selective sweep analysis between two populations always used allele configurations such as XP-CLR and XP-EHH, which focus on allele frequency or the haplotype blocks of variants. These methods were compared with two populations that showed differential evolution in the genomic regions (Chen et al. 2010; Manunza et al. 2016; Sun et al. 2018). Although XP-CLR and XP-EHH only consider the allele configuration and determine the significant genomic regions and encompassing genes, neither method can use genetic marker effects, as can be seen in GWA tests while determining the significant regions related to targeted phenotypes (Chen et al. 2010; Pritchard et al. 2010). Both selective sweep methods consider the hitchhiking effect around the target genomic regions (Santiago and Caballero 2005). Thus, if a specific genomic region is closely related to an interesting phenotype but without the hitchhiking effect, these methods could not detect genomic regions where two populations could be distinguished. However, XP-PAV analysis uses not only the evolutionary aspects based on the marker constitutions but also the marker effects related to the phenotype as a GWA test result. Because the different marker effects between the two populations can be an important basis for reflecting the characteristics of each population after domestic breeding, we suggest XP-PAV as a novel and simple method that could consider selective signal and marker effects for phenotypes. We believe that this method could detect the genomic differences between two populations contributing to the targeted phenotypes. We applied XP-PAV test to large-scale LL and YY population data in this study.

To obtain the statistics in XP-PAV analysis, we used the t-test after the GWA test and allele frequency calculation of the individual populations, which could assess the mean difference between the two population data (Ross and Willson 2017). Therefore, we thought that the t-test could be an adequate statistical test to uncover the differential effects of markers between the LL and YY populations. To select significant genetic variants in the XP-PAV analysis, we used an empirical *p*-value based on the t-value after standardization (z distribution), instead of the *p*-value from the t-test. Because this method was novel analysis, we calculated the *p*-value for the actual observed data instead of the theoretical data.

The genes of interest in the XP-PAV analysis for BF were growth hormone receptor (GHR; XP-PAV BF *p*-value 0.05), mechanistic target of rapamycin kinase (MTOR; XP-PAV BF *p*-value 0.05), and solute carrier family member 1 (SLC27A1; XP-PAV BF *p*-value 0.0001). It has been demonstrated that porcine growth hormone (pGH) increases muscle growth markedly, improves feed efficiency and protein synthesis (Yu-Jiang et al. 2020). GHR is involved in the JAK-STAT signaling pathway. In mammals, the JAK-STAT pathway is the major signaling mechanism for a variety of cytokines and growth factors in pigs (Wang et al. 2018). The mTOR gene encodes the mammalian target of rapamycin (mTOR), which regulates multiple biological processes such as growth and survival in response to hormones, growth factors, nutrients, energy, and stress signals as a serine/threonine protein kinase (Hay and Sonenberg 2004; Guertin and Sabatini 2007). SLC27A1 is positively correlated with intramuscular fat content. It is a long-chain fatty acid membrane transporter gene that is active in many cell types and is highly expressed in the gluteus medius, diaphragm, longissimus dorsi, and heart muscles (Gallardo et al. 2013; Wang et al. 2020). RUNX family transcription factor 1 (RUNX1) in the DWF analysis result was a notable gene. RUNX1 (located on SSC13, 140.0 Mb) is a candidate gene for this region based on transcription factor identification by promoter sequence analysis. Its protein product forms a heterodimer with CBFB, which binds to a number of enhancers and promoters, including murine leukemia virus, polyomavirus enhancer, LCK, IL-3, GM-CSF promoters, and T-cell receptor enhancers in pigs (Reiner et al. 2014).

Based on the results of the GO analysis, we found that the genetic and phenotypic differences were related to immune response (in DWG analysis), lipid metabolism, and protein modification process (in BF analysis) (Tables 2 and 3). In particular, ALOX15 (arachidonate 15-lipoxygenase), one of the genes in GO analysis, belongs to the glycerophospholipid biosynthetic (GO: 0046474) and protein modification processes (GO: 0036211). ALOX15 encodes a member of the lipoxygenase family of proteins and acts on various polyunsaturated fatty acid substrates to generate bioactive lipid mediators such as heparin, lipoxins, and eicosanoids. MAP3K7 (Mitogen-activated protein kinase 7) was related to positive regulation of interleukin-2 production (GO: 0032743), which mediates the signal transduction induced by TGF-β and morphogenetic protein (BMP) and controls a variety of cell functions, including transcription regulation and apoptosis (www.genecards.org). MAP3K7 was reported to be associated with growth traits (Hong et al. 2020).

## Conclusion

We suggested a XP-PAV considering the marker effect and allele configuration. XP-PAV analysis is a novel method compared to other methods that deal with signals of selection such as XP-EHH and XP-CLR. In this study, we performed XP-PAV analysis for the phenotypes DWG and BF in the LL and YY populations. In the XP-PAV analysis results, we found that the genetic differences between the two populations were closely related to the immune response, lipid metabolism, and protein modification process. In addition, we expect that the XP-PAV analysis could contribute to determining signals of selection in the future as a novel method for identifying genetic differences between two targeted populations.

## Data Availability

The datasets analyzed during the current study are not publicly available due to intellectual property considerations but are available from the corresponding author upon reasonable request.
